# Risk Alleles for Multiple Myeloma Susceptibility in ADME Genes

**DOI:** 10.3390/cells11020189

**Published:** 2022-01-06

**Authors:** Francesca Scionti, Giuseppe Agapito, Daniele Caracciolo, Caterina Riillo, Katia Grillone, Mario Cannataro, Maria Teresa Di Martino, Pierosandro Tagliaferri, Pierfrancesco Tassone, Mariamena Arbitrio

**Affiliations:** 1 Institute of Research and Biomedical Innovation (IRIB), Italian National Council (CNR), 98164 Messina, Italy; francesca.scionti@irib.cnr.it; 2 Department of Law, Economics and Sociology, Magna Graecia University of Catanzaro, 88100 Catanzaro, Italy; agapito@unicz.it; 3 Data Analytics Research Center, Magna Graecia University of Catanzaro, 88100 Catanzaro, Italy; cannataro@unicz.it; 4 Department of Experimental and Clinical Medicine, Magna Graecia University, 88100 Catanzaro, Italy; d.caracciolo@unicz.it (D.C.); caterina.riillo1@studenti.unicz.it (C.R.); k.grillone@unicz.it (K.G.); teresadm@unicz.it (M.T.D.M.); 5 Department of Medical and Surgical Sciences, Magna Graecia University of Catanzaro, 88100 Catanzaro, Italy; 6 Institute of Research and Biomedical Innovation (IRIB), Italian National Council (CNR), 88100 Catanzaro, Italy

**Keywords:** multiple myeloma, single nucleotide polymorphism, SNP, ADME, risk alleles, hematological malignancies, DMET Plus

## Abstract

The cause of multiple myeloma (MM) remains largely unknown. Several pieces of evidence support the involvement of genetic and multiple environmental factors (i.e., chemical agents) in MM onset. The inter-individual variability in the bioactivation, detoxification, and clearance of chemical carcinogens such as asbestos, benzene, and pesticides might increase the MM risk. This inter-individual variability can be explained by the presence of polymorphic variants in absorption, distribution, metabolism, and excretion (ADME) genes. Despite the high relevance of this issue, few studies have focused on the inter-individual variability in ADME genes in MM risk. To identify new MM susceptibility loci, we performed an extended candidate gene approach by comparing high-throughput genotyping data of 1936 markers in 231 ADME genes on 64 MM patients and 59 controls from the CEU population. Differences in genotype and allele frequencies were validated using an internal control group of 35 non-cancer samples from the same geographic area as the patient group. We detected an association between MM risk and *ADH1B* rs1229984 (OR = 3.78; 95% CI, 1.18–12.13; *p* = 0.0282), *PPARD* rs6937483 (OR = 3.27; 95% CI, 1.01–10.56; *p* = 0.0479), *SLC28A1* rs8187737 (OR = 11.33; 95% CI, 1.43–89.59; *p* = 0.005), *SLC28A2* rs1060896 (OR = 6.58; 95% CI, 1.42–30.43; *p* = 0.0072), *SLC29A1* rs8187630 (OR = 3.27; 95% CI, 1.01–10.56; *p* = 0.0479), and *ALDH3A2* rs72547554 (OR = 2.46; 95% CI, 0.64–9.40; *p* = 0.0293). The prognostic value of these genes in MM was investigated in two public datasets showing that shorter overall survival was associated with low expression of *ADH1B* and *SLC28A1*. In conclusion, our proof-of-concept findings provide novel insights into the genetic bases of MM susceptibility.

## 1. Introduction

Multiple myeloma (MM) accounts for approximately 1% of all cancers and 10% of all hematologic malignancies. MM remains a common and incurable disorder, although remarkable progress in treatment has been made [1]. In 2020, based on the GLOBOCAN database (https://gco.iarc.fr/today, accessed on 10 November 2021 ), the estimated number of new cases diagnosed yearly in Europe was over 50,000, and almost 32,000 patients die of the disease. There is strong evidence that obesity, systemic inflammation, oxidative stress, and exposure to radiation or environmental chemical agents might increase the risk of MM [2]. Moreover, it has been also reported that inherited genetic variants significantly contribute to MM onset, accounting for approximately 16% of cases [3]. 

Environmental exposure to chemicals such as dioxin, asbestos, benzene, and pesticides are causally connected to MM transition from the two stages of Monoclonal Gammopathy of Uncertain Significance (MGUS) and Smouldering Multiple Myeloma [4]. The metabolism of chemical carcinogens relies on key processes such as bioactivation by phase I drug-metabolizing enzymes, detoxification by phase II drug-metabolizing enzymes, and elimination by transporters. The genes encoding these proteins, known as Absorption, Distribution, Metabolism, Excretion (ADME) genes, are highly polymorphic and their role is also well established in the metabolism of endobiotics (i.e., hormones, steroids, and bile acids) and in pharmacogenetics, where the genetic profile of relevant ADME genes is used to guide tailored prescription and dose adjustments [5,6]. 

The inter-individual variability in cancer susceptibility, including in MM, may be partially explained by genetic variations in xenobiotic enzymes that can enhance or impair the transformation of chemicals into active carcinogens. On this basis, there is an urgent need of studies focused to the understanding of the effective role of chemical carcinogens in MM risk. So far, some studies have investigated the association of single nucleotide polymorphisms (SNPs) in ADME genes with MM risk [7,8], but no studies have explored this relationship using an extended candidate gene approach. Therefore, the toxic role in MM onset needs more efforts for disclosing its real impact in MM risk. 

In this scenario, we simultaneously genotyped 1936 tagSNPs in 231 ADME genes using the DMET^TM^ SNP panel to identify unknown potential biomarkers associated with MM susceptibility in 64 MM patients compared to 59 healthy individuals from the CEU population to investigate the presence of differences in allele frequencies and the association to MM onset risk. Differences in genotype and allele frequencies between cases and controls were validated using an internal control group of 35 non-cancer samples from the same geographic area as the patient group.

## 2. Materials and Methods

### 2.1. Sample Collection and Genotyping

A total of 64 MM patients at diagnosis were enrolled from February 2012 to June 2017 at the Medical and Translational Oncology Unit of Mater Domini Hospital of Magna Graecia University, Catanzaro, Italy. The study was compliant with Institutional bioethical standards and each individual provided a standard informed consent and agreed to use his/her biological sample for research purposes. 

Peripheral blood (3 mL) from each patient was collected for genomic DNA extraction using the Perfect Pure DNA Blood kit (5 Prime) and according to the manufacture’s instruction. Genotyping of 1936 ADME markers was performed using the DMET Plus assay (Thermo Fisher Scientific, Waltham, MA, USA.) as previously described [9,10,11]. Genotypes were extracted using DMET Console software version 1.1 (Thermo Fisher Scientific, Waltham, MA, USA). A call rate ≤ 96%, 100% of genotype concordance among the two groups and high rate of “possible rare allele”/“no call” as exclusion criteria from further analysis was used. Allele frequency values for the 59 CEU Hapmap samples are available on GPL17860 dataset. The internal dataset including 35 non-cancer samples was previously genotyped with the same platform and previously topic of two publications [12,13]. 

### 2.2. Cross-Validation Leave-One-Out Analysis

We carried out internal validation in our MM dataset by a Cross Validation (CV)-based approach, called k-fold CV. We split the dataset into k equal partitions (or folds). The k partitions are splitted in “test” and “training” datasets, to train and test the performance measure of the exact selection of MM risk alleles identified by DMET-Analyzer. We used the first fold as the test set and the union of the other folds as the training set, and we computed the accuracy repeating these steps ten times (K = 10). 

### 2.3. Overall Survival Analysis

We investigated genes with *p*-value ≤ 0.05 to identify any correlation with patients’ prognosis. We performed overall survival (OS) analysis using the survival analysis function available on the GenomicScape online web tool (http://www.genomicspace.com, accessed on 21 April 2021). The first dataset includes 414 untreated MM patients from the University of Arkansas (GSE4581) analyzed by the Affymetrix GeneChip™ Human Genome U133 Plus 2.0 Array while the second cohort includes 264 relapsed untreated MM patients (GSE9782) from Mulligan et al. [14] analyzed by the Affymetrix GeneChip™ Human Genome U133 A/B. All probe sets represented on the GeneChip™ Human Genome U133A/B Array were identically replicated on the GeneChip Human Genome U133A 2.0 Array. In Kaplan–Meier curves, generated using GenomicScape web tool, the optimal cut-off for dividing expression in “high” or “low” is estimated automatically using the Maxstat cutpoint threshold algorithm, foregone the data normalization performed using the variance stabilizing normalization algorithm.

### 2.4. Statistical Analysis

Comparison of genotype frequency in the MM patients versus CEU population was performed by Fisher’s exact test [15]. Bonferroni’s correction was applied for multiple comparisons. Hardy-Weinberg equilibrium in the control group was tested using χ^2^ test. Results of potential interest were limited to those in which the *p*-value was ≤0.05. Multiple logistic regression models (over-dominant and recessive) were employed to calculate odds ratios (ORs), 95% confidence intervals (CIs), and *p*-values using Med Calc v12.3.0. 

In OS analysis, only statistically significant results showing a concordant trend by probe set between the two datasets were considered. Hardy-Weinberg equilibrium was tested for each polymorphic variant.

### 2.5. Pathway Enrichment Analysis

Pathway enrichment analysis (PEA) helps to obtain mechanistic awareness into gene lists produced from genomics investigations and identifies biological pathways enriched in a gene list. PEA includes three essential steps: (i) definition of a genes list of interest from the disease under investigation; (ii) determination of statistically enriched pathways performed using a BioPAX-Parser (BiP) software tool [16]; (iii) interpretation of enriched pathways to identify the primary biological relationships among genes of interest and enriched pathways in the dataset. To perform the enrichment analysis with BiP is necessary to provide a list of proteins or genes of interest and select a pathway database. We used the genes listed in Table 1 that exhibited a *p*-value < 0.05 computed using the DMET-Analyzer software tool and using the Reactome pathways databases. BiP is used with the default options: significance threshold of 0.05 for *p*-value, and the whole homo sapiens pathway as the reference background. We considered a pathway significantly enriched by BiP if its p-value is smaller than 0.05 after applying hypergeometric and multiple hypotheses testing corrections with the FDR and Bonferroni correctors. 

An enrichment result could help identify interesting pathways not previously associated with the experimental context, for which a more careful evaluation is necessary to be validated as potential discoveries [17].

## 3. Results

### 3.1. SNPs Associated to MM Risk

Among all SNPs in DMET Plus, 738 met our criteria. From statistical analysis, we identified an association between MM risk and the *SLC28A2* rs1060896 (*p* = 0.0125) while we found that the Minor Allele Frequency (MAF) for seven variant alleles in seven genes was undetectable in the CEU control group and over-represented in the MM group. Specifically, the alleles A for *ADH1B* rs1229984, A for *PPARD* rs6937483, A for *POR* rs2286824, A for *SLC29A1* rs8187630, T for *SLC28A1* rs8187737, T for *ALDH3A2* rs72547554 and, G for *SLC19A1* rs60881836 were absent in CEU group. To verify possible differences in population structure set-up, we compared MM genotype data with an internal control group. We confirmed the association between rs1060896 and MM risk (*p* = 0.0072) and found that 5/7 alleles (T for *ALDH3A2* rs72547554, A for *SLC29A1* rs8187630, T for *SLC28A1* rs8187737, A for *PPARD* rs6937483, A for *ADH1B* rs1229984) are also present in our internal dataset, although at a very low frequency, but significantly associated with MM risk (Table 1). Instead, the A allele for *POR* rs2286824 and *SLC19A1* rs60881836 remained absent and thus excluded from further analysis. The test performance to verify the generalization power of our model using an independent dataset has been obtained using a CV leave-one-out–based method. Using a training set different from the test set, we estimated the accuracy of the selected SNPs from the MM dataset reaching an accuracy of 94.99%.

### 3.2. Correlation with OS

**Figure 1:**
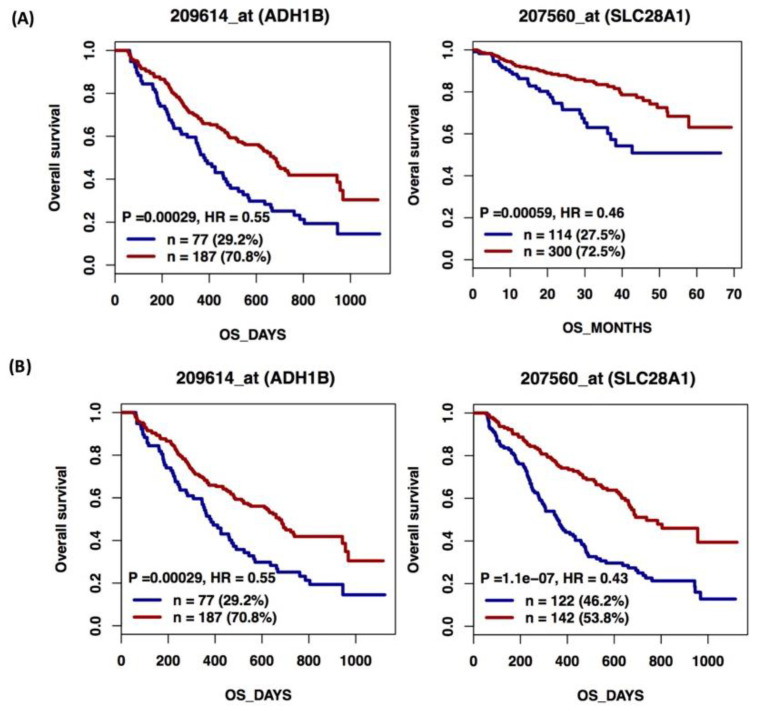
Overall survival (OS) by gene expression level. The survival analysis of 414 MM patients from the Arkansas cohort in GSE4581 (**A**) and 264 MM patients in GSE9782 (**B**) from Mulligan et al., classified according to gene expression levels: high (red) or low (blue). We used days’s scale to plot OS. P: *p*-value; HR: Hazard Ratio; n: number of patients.

To evaluate if these ADME genes might have a prognostic role in MM disease outcome, in terms of OS, we analyzed their expression in two independent public datasets of MM patients, GSE4581 and GSE9782, available on GenomicScape. Survival analysis showed that in MM patient datasets shorter OS was associated with low expression of *ADH1B* and *SLC28A1* (Figure 1A,B).

### 3.3. Pathway Enrichment Results

**Figure 2:**
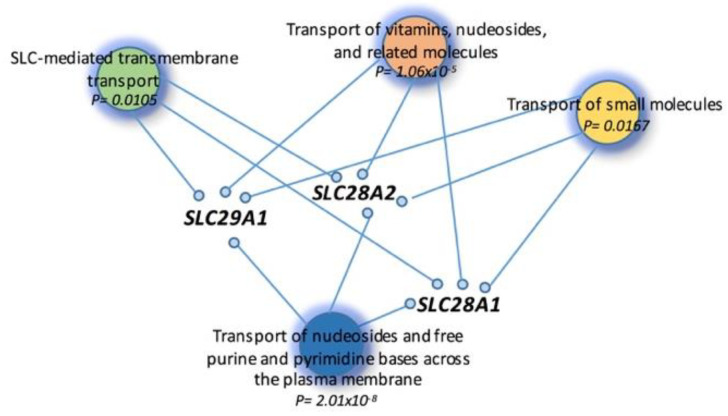
Inter-connection of metabolic pathways. Pathway enrichment analysis highlights four pathways in which three genes are mainly involved. P: *p*-value.

Using the gene list and Reactome database, BiP was able to enrich a total of 14 relevant pathways with respect to the MM. *PPARD* is involved in nine important pathways: “RA biosynthesis pathway” (*p* = 0.0064), “The canonical retinoid cycle in rods” (*p* = 0.0122), “Nuclear Receptor transcription pathway” (*p* = 0.0177), “Metabolism of fat-soluble vitamins” (*p* = 0.0232), “Intracellular metabolism of fatty acids regulates insulin secretion” (*p* = 0.0271), “Carnitine metabolism” (*p* = 0.0277), “Regulation of pyruvate dehydrogenase (PDH) complex” (*p* = 0.0344), “Phase I-Functionalization of compounds” (*p* = 0.0370) and “Signaling by Retinoic Acid” (*p* = 0.0426). *ADH1B* is involved in “Ethanol oxidation” (*p* = 0.0027). Figure 2 shows the interaction network among the selected genes into the metabolic pathway.

## 4. Discussion

The complexity of the interplay between environment and genes in cancer development can be affected by genetic variants. Products of endobiotic/xenobiotic metabolism can in fact impact the onset and progression of cancer by the activation of DNA damage response and growth factor signaling pathways. Inter-individual variability in cancer susceptibility could be partially explained by genetic polymorphisms in ADME genes that, by altering gene expression or protein function, may affect key metabolic pathways involved in xenobiotic biotransformation [18]. In this study, we investigated the correlation between polymorphic variants in ADME genes and the MM risk by a high-throughput genotyping platform. By DMET analyzer, we found differences in genotype distribution between MM and CEU in eight SNPs. In particular, the homozygous genotype for the ancestral allele in *SLC28A2* rs1060896 showed association with MM risk, while seven variant alleles were absent in CEU control group. To verify if the observed allele distributions were due to a different genomic structure among the two groups, we used previously published DMET genotype data from a group of 35 non-cancer patients. With this analysis, we confirmed the association between rs1060896 and MM risk and verified the presence of five out of seven variant alleles in our internal dataset at a very low frequency (T rs72547554, MAF = 0.043; A rs8187630, MAF = 0.057; T rs8187737, MAF = 0.014; A rs6937483, MAF = 0.057; A rs1229984, MAF = 0.114). The A allele for *POR* rs2286824 and G allele for *SLC19A1* rs6088183 were absent, probably due to a very low population frequency. These findings did not replicate previous genomic loci identified in several studies as related to MM risk. A possible explanation might be the occurrence of population differences. In fact, the risk alleles highlighted in our population have very low frequencies that have not been reported in any database so far. However, the impact of risk alleles identified in this study needs further evaluation and validation in an independent cohort of MM patients as well as cross-platform replication. So far, only the functional rs1229984 in *ADH1B* (Alcohol Dehydrogenase 1B (Class I), Beta Polypeptide), characterized by a G > A transition with an amino acidic change from arginine (Arg) to histidine (His), has been reported to be associated with cancer risk among Caucasians (increased risk, A allele MAF ≥ 0.03) and Asians (decreased risk, A allele MAF ≥ 0.3). The His/His individuals have a 40-fold higher oxidative activity of ethanol to toxic acetaldehyde compared to Arg/Arg individuals [19]. Acetaldehyde is an important carcinogen for its highly toxic effects due to interference with many biological processes including DNA synthesis. In this study, the expression of two of the investigated genes showed a significant association with OS in MM public datasets, suggesting a potential involvement in MM progression. By analyzing the GSE4581 and GSE9782 datasets, we found a positive correlation between the high expression of *ADH1B* and *SLC28A1* and OS. *SLC28A1*, encodes for a nucleoside transporter primarily involved in the cellular uptake of nucleosides and nucleoside analogs such as gemcitabine and 5-fluorouracil. In pancreatic cancer, *SLC28A1* was down-regulated when compared with non-neoplastic tissues [20], and high tumor transcript levels of *SLC28A1* predicted a poor OS in pancreatic ductal adenocarcinomas patients. In addition, according to our *proof-of-concept* hypothesis, we found, using a functional pathway enrichment analysis, that the activity of the protein encoded by these genes are involved in 14 important and potential carcinogenic pathways. In particular, the solute carrier genes (*SLC28A1*, *SLC28A2*, *SLC29A1*) share four common pathways. In this context, it is important to highlight that the genetic interaction bridging pathways often connect genes that may contribute to a specific phenotype not always due to the effect of a single gene [21]. Several reports provided evidence of the role of others among these genes in cancer risk. For instance, the high expression of *ALDH3A2* correlated with low-grade and longer OS in gastric carcinoma patients [22]. The ALDH3A2 enzyme is involved in the detoxification of aldehydes generated by alcohol metabolism and lipid and fatty acids peroxidation, key steps for ATP production. For *PPARD* (Peroxisome proliferator-activated receptor-δ) encoding a nuclear transcriptional receptor, there is evidence of its up-regulation in several major human cancers, including colorectal, pancreatic, and lung cancer. Overall, these findings, although preliminary, provide novel information into the inherited susceptibility to MM. The integration of multi-omics data from validated approaches at different molecular levels could provide significant understanding of the complexity of biological systems [23]. To our knowledge, this is the first application of a broad multi-gene panel approach to investigate the correlation of polymorphic variants in ADME genes with MM risk. However, the major limitations of the current study were the small sample size, the lack of validation of our findings in an independent population, OS analysis limited to gene expression values and not to genotype classes, and the lack of functional studies in these SNPs and the absence of direct mechanistic biological insights. In conclusion, our *proof-of-concept* study provides preliminary evidence of the involvement of these variants as genetic indicators of MM risk and needs validation in a larger, independent group and other populations.

## Figures and Tables

**Table 1:** Summary results for SNPs associated with MM risk.

Gene (Location) dbSNP	MAF MM	MAF CEU	MAF ID	Genotype	MM (*n* = 64)	CEU (*n* = 59)	ID (*n* = 35)	*p*-Value MM vs. CEU	*p*-Value MM vs. ID	OR 95% C.I. MM vs. CEU	OR 95% C.I. MM vs. ID	Model
*SLC28A2* (15q21.2) rs1060896	C = 0.445	C = 0.250	C = 0.314	CC AC AA	15 27 22	4 24 31	2 18 15	0.0125	0.0072	4.21, 1.31 to 13.54	6.58, 1.42 to 30.43	Recessive
*ADH1B* (4q23) rs1229984	A = 0.195	A = 0.000	A = 0.114	GG AG AA	41 21 2	59 0 0	29 4 2	-	0.0282	-	3.78, 1.18 to 12.13	over-dominant
*PPARD* (6p21.31) rs6937483	A = 0.148	A = 0.000	A = 0.057	GG AG AA	45 19 0	59 0 0	31 4 0	-	0.0479	-	3.27, 1.0143 to 10.56	over-dominant
*POR* (7q11.23) rs2286824	A = 0.164	A = 0.000	A = 0.000	GG AG AA	43 21 0	59 0 0	35 0 0	-	-	-	-	-
*ALDH3A2* (17p11.2) rs72547554	T = 0.094	T = 0.000	T = 0.043	CC CT TT	52 12 0	59 0 0	32 3 0	-	0.0293	-	2.46, 0.6448 to 9.40	over-dominant
*SLC19A1* (6p21.1) rs60881836	G = 0.148	G = 0.000	G = 0.000	GG AG AA	0 19 45	0 0 59	0 0 35	-	-	-	-	-
*SLC29A1* (21q22.3) rs8187630	A = 0.148	A = 0.000	A = 0.057	GG AG AA	45 19 0	59 0 0	31 4 0	-	0.0479	-	3.27, 1.0143 to 10.56	over-dominant
*SLC28A1* (15q25.3) rs8187737	T = 0.125	T = 0.000	T = 0.014	CC CT TT	48 16 0	59 0 0	34 1 0	-	0.005	-	11.33, 1.43 to 89.59	over-dominant

dbSNP: SNP identifier based on NCBI; ID: Internal Dataset; OR: Odds Ratio; C.I.: confidence interval.

## Data Availability

The datasets presented in this study can be found in online repositories. The names of the repositories and accession numbers can be found below: https://www.ncbi.nlm.nih.gov/geo/, GSE187009, accessed on 03 November 2021; https://www.ncbi.nlm.nih.gov/geo/, GPL17860, accessed on 11 March 2021; https://www.ncbi.nlm.nih.gov/geo/, GSE4581, accessed on 21 April 2021; https://www.ncbi.nlm.nih.gov/geo/, GSE9782, accessed on 21 April 2021.
